# MicroED: conception, practice and future opportunities

**DOI:** 10.1107/S2052252521013063

**Published:** 2022-01-18

**Authors:** Max T. B. Clabbers, Anna Shiriaeva, Tamir Gonen

**Affiliations:** a Department of Biological Chemistry, University of California, Los Angeles, CA 90095, USA; b Howard Hughes Medical Institute, University of California, Los Angeles, CA 90095, USA; c Department of Physiology, University of California, Los Angeles, CA 90095, USA

**Keywords:** MicroED, cryo-EM, microcrystal electron diffraction, crystallography, membrane proteins

## Abstract

This article documents a keynote seminar presented at the IUCr Congress in Prague, 2021. The cryo-EM method microcrystal electron diffraction is described and put in the context of macromolecular electron crystallography from its origins in 2D crystals of membrane proteins to today’s application to 3D crystals a millionth the size of that needed for X-ray crystallography. Milestones in method development and applications are described with an outlook to the future.

## Abstract 2

Microcrystal electron diffraction is described and put in the context of its origins in macromolecular crystallography.

## 1. Electron cryomicroscopy

**Figure 1:**
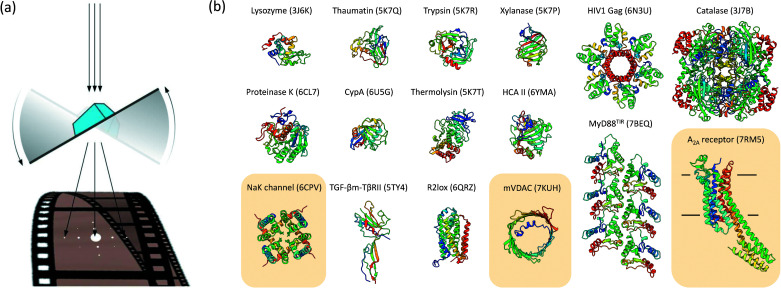
The MicroED method and examples of some structures. (*a*) Illustration of MicroED data collection where the crystal is continuously rotated and diffraction patterns are recorded as a movie. (*b*) Examples of several protein structures determined by MicroED are listed with their names and PDB identifiers. Proteins are shown in their biological assembly and membrane proteins are highlighted in yellow.

**Figure 2:**
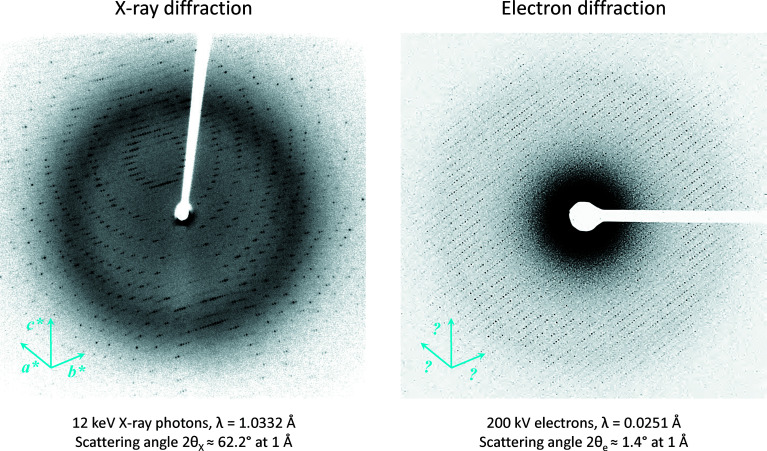
Differences between X-ray and electron diffraction. Diffraction patterns are shown for X-ray diffraction and MicroED. The much shorter wavelength of high-energy electrons means that the Ewald sphere construction is virtually flat. Indexing from a single diffraction frame is therefore not typically possible for electron diffraction, and a larger wedge of reciprocal space needs to be covered to find the unit-cell dimensions.

**Figure 3:**
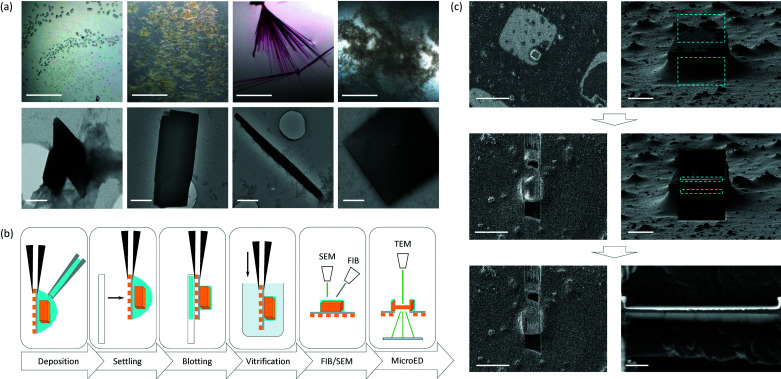
Sample preparation for macromolecular MicroED. (*a*) Several examples of crystallization conditions with protein microcrystals of different morphologies suitable for MicroED. Crystals can be identified using light microscopy as shown in the top row, corresponding images of negatively stained samples are shown on the bottom row. Scale bars for the top row are 500 nm and bottom row 400 nm. Images were adapted from the work by Shi *et al.* (2016 ▸). (*b*) Typical workflow involved in MicroED sample preparation for protein crystals. The crystals are pipetted from the crystallization drop and deposited onto the carbon-side of a glow-discharged EM-grid held between the tips of a tweezer. The crystals are allowed to settle on the grid before any excess liquid is blotted away from the back-side of the grid with filter paper. The grid is then rapidly plunged into liquid ethane for freezing and kept at cryogenic temperatures until use. The grid is either transferred directly to the TEM for MicroED data collection, or can be thinned by cryo-FIB milling into crystalline lamellae suitable for MicroED. (*c*) FIB milling of tetragonal lysozyme crystals, showing the SEM images (left) side by side with the FIB images (right) during a typical milling workflow as described previously by Martynowycz & Gonen (2021*a* ▸). After a suitable crystal is identified, rectangular boxes (blue) are drawn for coarse milling the bulk material (top). The resulting 3 µm-thick lamella is then further thinned by polishing using a lower current and smaller step sizes (middle). This results in a thin crystalline lamella of ideally 200–300 nm thickness and a width of 5 µm (bottom). Scale bars for the SEM images from top to bottom are 50, 25 and 25 µm, for the FIB images from top to bottom 10, 10 and 1 µm.

**Figure 4:**
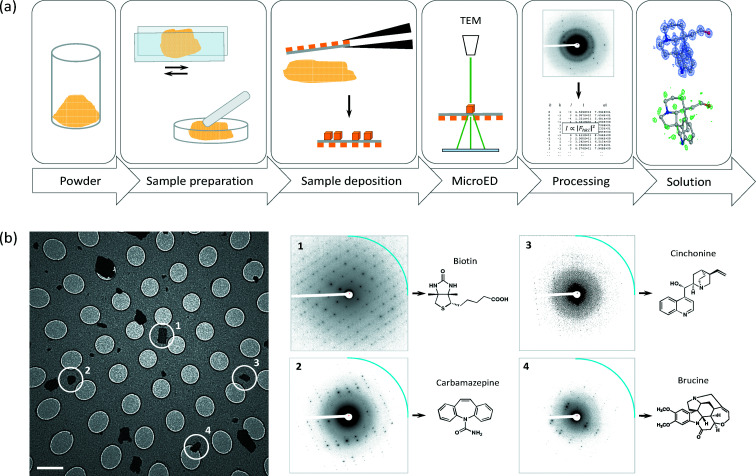
MicroED for small-molecule research. (*a*) Typical workflow involved for MicroED of small-molecule samples from powder to individual crystals on a standard EM grid, data collection, processing and structure solution. The solved structure in the last panel shows the model of the compound limaspermidine with the observed map showing the individual atoms (blue, top), and the difference map showing hydrogen atom positions (green, bottom). (*b*) Individual compounds can be identified from heterogeneous mixtures using MicroED. Resolution rings are shown at 0.8 Å (blue).

**Figure 5:**
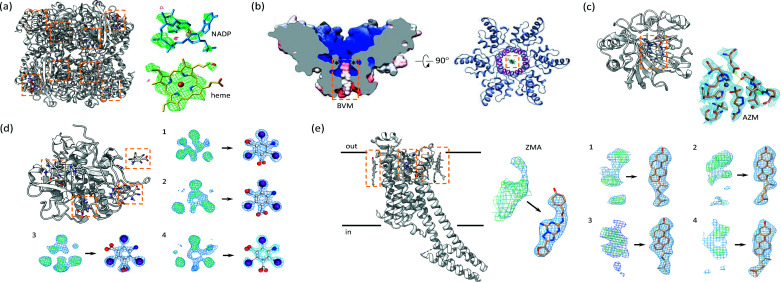
MicroED and drug discovery. (*a*) Ligand binding showing the heme group and NADP resolved by MicroED for bovine liver catalase (Nannenga, Shi, Hattne *et al.*, 2014 ▸). (*b*) Drug binding of the inhibitor bevirimat (BVM) in complex with the C-terminal domain of the HIV Gag protein fragment (Purdy *et al.*, 2018 ▸). (*c*) Drug-bound MicroED structure of human carbonic anhydrase isoform II (HCA II) complexed with the clinical drug acetazolamide (AZM) (Clabbers *et al.*, 2020 ▸). Inset shows the active site where the ligand is coordinated to the active site zinc metal co-factor. (*d*) Efficient ligand soaking into microcrystals was demonstrated from lamellae of proteinase K that were briefly soaked on-grid with I3C compounds (Martynowycz & Gonen, 2021*a* ▸). Four I3C molecules could be identified in the difference map, each difference map is shown next to the observed map of the fitted and refined ligands. (*e*) Structure of the adenosine A_2A_-receptor determined from LCP crystals using MicroED (Martynowycz, Shiriaeva *et al.*, 2021 ▸). The ligand ZM241385 (ZMA) could be resolved in the orthosteric pocket, as well as four surrounding cholesterol molecules bound to the receptor on the extracellular side. Difference maps are shown next to the refined maps with the fitted ligand and the four numbered cholesterol molecules. In all panels, the positions of the ligand in the protein models are highlighted with yellow rectangular boxes.

**Figure 6:**
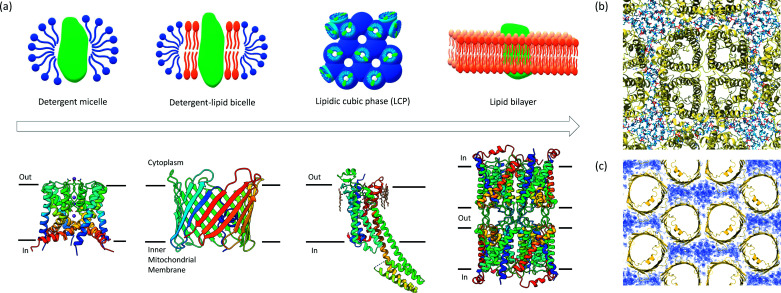
Examples of membrane protein crystallization modalities and structures solved. (*a*) Membrane protein structure determination in MicroED in a lipid and/or detergent environment mimicking the lipid bilayer. The structure of the NaK ion channel was determined from detergent micelles (Liu & Gonen, 2018 ▸), mVDAC from lipid bicelles (Martynowycz *et al.*, 2020 ▸), the A_2A_-receptor from LCP crystals (Martynowycz, Shiriaeva *et al.*, 2021 ▸), whereas aqua­porin-0 (AQP0) was determined from double-layered crystals of the protein embedded in lipid bilayers using electron diffraction (Gonen *et al.*, 2005 ▸). (*b*) Top view showing the electron crystallography structure of AQP0 resolved lipid-protein packing interactions between the tetramer (yellow) and the surrounding lipid molecules (blue). (*c*) Top view showing the MicroED structure of mVDAC and the crystal packing of the monomers (yellow) where the map (blue) in between the barrels indicates the space is likely to be occupied by the lipid molecules.

Electron cryomicroscopy (cryo-EM) is an important technique in structural biology. Electrons are scattered very efficiently by the sample, at the cost of only a modest amount of radiation damage, compared with X-rays and neutrons (Henderson, 1995 ▸). In cryo-EM, electrons are used to probe the underlying structure of vitrified biological specimens using a transmission electron microscope (TEM). In cryo-EM imaging-based techniques, the electrons scattered by the sample are focused into a real-space image. In single-particle cryo-EM, high-resolution images are recorded of many individual protein complexes captured in random orientations (Cheng, 2015 ▸). These 2D projection images can be combined in Fourier space based on their various angular contributions to reconstruct a 3D real space model (De Rosier & Klug, 1968 ▸). In electron tomography the specimen is tilted and images are taken at each discrete tilt angle, and a 3D structural model is obtained that is similar to the reconstruction method in single-particle analysis (Li, 2021 ▸). In cryo-EM diffraction-based techniques, the electron wave scattered by the sample is measured directly in reciprocal space. The specimen is an ordered crystalline array of biomolecules, such that the coherently diffracted electrons by the crystal are focused into Bragg spots of the reciprocal lattice. Although the signal-to-noise ratio is typically better in diffraction, imaging has the main advantage that the spatial phase information – lost in diffraction – is retained. In electron crystallography, 2D protein crystals are typically studied by combining imaging and diffraction (Glaeser & Downing, 1993 ▸; Fujiyoshi, 1998 ▸). Here, an initial 3D structural model of the protein is reconstructed using phases extracted from the Fourier transforms of images, which are then extended to higher resolution using intensities obtained from electron diffraction patterns (Wisedchaisri & Gonen, 2011 ▸). More recently, membrane protein structures could be determined from layered 2D crystals solely using diffraction combined with phases from molecular replacement (Gonen *et al.*, 2004 ▸, 2005 ▸). Similarly, in microcrystal electron diffraction (MicroED), diffraction from small 3D crystals is used together with molecular replacement or *ab initio* methods for structure determination (Figs. 1–6) (Shi *et al.*, 2013 ▸; Nannenga, Shi, Leslie *et al.*, 2014 ▸; Martynowycz, Clabbers, Hattne & Gonen, 2021 ▸).

## 2. Electron crystallography of 2D crystals

Working with biological specimens in cryo-EM requires that the proteins are preserved in their native hydrated state and that any sample irradiation is minimal or very brief to preserve the structural integrity of the protein. These are primarily the differences between cryo-EM and non-cryogenic methods that are typically used for radiation-hard metals and materials research. Initial electron microscopy studies in structural biology focused on large 2D protein crystals or virus particles that were typically stabilized by embedding, or fixed by staining, to withstand the vacuum and exposure dose in the electron microscope. For example, bovine liver catalase was extracted and purified as early as the 1930s and was observed to assemble into thin crystalline plates (Sumner & Dounce, 1937 ▸). In 1968, De Rosier & Klug (1968 ▸) published their landmark work introducing the 3D reconstruction method to calculate a density map of the bacteriophage *T*4 tail using Fourier transforms of electron microscope images. The first demonstration of 2D electron crystallography showed that high-resolution electron diffraction patterns could be collected from hydrated catalase crystals of 4.5 nm thickness using a hydration stage without staining or fixation (Matricardi *et al.*, 1972 ▸). Taylor & Glaeser (1974 ▸) demonstrated the first use of electron cryomicroscopy by freezing catalase crystals, preserving the specimen in its native hydrated state during electron diffraction data collection. Dorset & Parsons (1975*a* ▸,*b* ▸) used a hydration stage to record high-resolution electron diffraction patterns from hydrated 3D microcrystals of catalase as thick as ∼150 nm.

Henderson and Unwin presented the first 3D structural models by electron crystallography from glucose-embedded 2D crystals of the purple membrane protein bacterio­rhodopsin and bovine liver catalase at 7 and 9 Å resolution, respectively (Henderson & Unwin, 1975 ▸; Unwin & Henderson, 1975 ▸). They used both imaging and diffraction, obtaining phases extracted from Fourier transforms of the images that were combined with intensities obtained from electron diffraction patterns to reconstruct a 3D density map (De Rosier & Klug, 1968 ▸; Unwin & Henderson, 1975 ▸). In 1984, Dubochet and co-workers introduced rapid sample vitrification, where biological specimens are plunged into liquid ethane for freezing while remaining hydrated in a thin layer of vitreous ice (Adrian *et al.*, 1984 ▸). In the following years, near-atomic resolution models were obtained of bacteriorhodopsin using electron crystallography of cryogenically preserved 2D crystals (Henderson *et al.*, 1990 ▸; Grigorieff *et al.*, 1996 ▸; Mitsuoka *et al.*, 1999 ▸). Other milestones in electron crystallography were structural models of the light-harvesting complex II (Kühlbrandt *et al.*, 1994 ▸), the alpha–beta tubulin heterodimer (Nogales *et al.*, 1998 ▸) and the water channel protein aqua­porin-1 (AQP1) (Walz *et al.*, 1997 ▸; Murata *et al.*, 2000 ▸). The structure of the acetyl­choline receptor in the open conformation was solved using time-resolved electron crystallography (Unwin, 1995 ▸).

The first protein solved at atomic resolution by cryo-EM was the structure of aqua­porin-0 (AQP0) at 1.9 Å resolution from double-layered crystals (Gonen *et al.*, 2005 ▸). Notably, the structure was solved by electron crystallography exclusively using electron diffraction patterns recorded at different tilt angles and phased by molecular replacement revealing the structure of the AQP0 tetramer, water molecules and the surrounding membrane (Fig. 6). Furthermore, it was shown that even when only initial low-resolution phases are available, these can be extended using high-resolution electron diffraction data to reconstruct atomic resolution models (Wisedchaisri & Gonen, 2011 ▸). Electron crystallography was also used to solve the structure of aqua­porin-4 (AQP4) from double-layered crystals (Hiroaki *et al.*, 2006 ▸), and the Connexin26 (Cx26) gap junction channel structure was determined from crystals of three layers (Oshima *et al.*, 2007 ▸, 2011 ▸). A major advantage of electron crystallography using single or multiple layers is that membrane proteins can be reconstituted in their native environment, enabling study of their functionally and interactions within the lipid bilayer (Fig. 6). However, crystallization and sample preparation of 2D-layered crystals is involved, and crystal defects and imperfections can limit the data quality and complicate reconstruction, especially for projection images at higher tilt angles (Glaeser & Downing, 1993 ▸). Care must be taken during sample preparation that these plate-like crystals are laid entirely flat on the EM grid, and the microscope should be set up accordingly for collecting electron diffraction data (Gonen, 2013 ▸). Limitations on imperfect crystals can be further mitigated using single-particle image processing routines to align and reconstruct patches of individual protein molecules within the bilayer selected from high-resolution images (Righetto *et al.*, 2019 ▸).

## 3. Microcrystal electron diffraction

In 2013, the structure of hen egg-white lysozyme was determined from a 3D crystal by microcrystal electron diffraction (MicroED) using still diffraction patterns recorded at discrete tilt steps (Shi *et al.*, 2013 ▸). This was the very first protein structure determined by electron diffraction from multilayered 3D crystals (the crystals had approximately ten layers of lysozyme). Soon after, the method was modified to include continuous rotation [Fig. 1 ▸(*a*)] (Nederlof, van Genderen *et al.*, 2013 ▸; Nannenga, Shi, Leslie & Gonen, 2014 ▸). This is analogous to the rotation method in macromolecular X-ray crystallography (MX), enabling for a near-complete sampling of reciprocal space using fine-slicing (Arndt & Wonacott, 1977 ▸; Pflugrath, 1999 ▸; Dauter, 1999 ▸). Using continuous rotation, it became possible to process MicroED data with X-ray processing programs (Nannenga, Shi, Leslie & Gonen, 2014 ▸), resulting in better quality data as the sampling of the reciprocal space was more fine, and the lysozyme structure was determined from a single microcrystal to higher resolution than reported previously. That same year, the structure of catalase was solved by MicroED at 3 Å resolution (Nannenga, Shi, Hattne *et al.*, 2014 ▸). This work used a single plate-like 3D microcrystal of approximately 150 nm thickness, consistent with earlier observations on hydrated catalase crystals (Dorset & Parsons, 1975*b* ▸). However, in earlier studies electron diffraction data collection was limited to recording a single high-resolution still diffraction pattern per crystal owing to radiation damage and because data collection was carried out on early electron microscopes using plate film, photographic film or old charge-coupled devices (Matricardi *et al.*, 1972 ▸; Taylor & Glaeser, 1974 ▸; Dorset & Parsons, 1975*a* ▸). Working with beam-sensitive biological specimens in cryo-EM, MicroED data are rapidly collected by continuously rotating cryo-cooled crystals under low-dose conditions (Shi *et al.*, 2016 ▸). For example, to illustrate the effects of radiation damage, 3D crystals of proteinase K exposed to the electron beam lose half of their mean diffracted intensities after a dose of about 2.2 e^−^ Å^−2^ (Hattne *et al.*, 2018 ▸). Furthermore, sample irradiation causes site-specific damage such as breaking of di­sulfides at a dose of about 0.9 e^−^ Å^−2^ and de­carboxyl­ation of acidic side chains at 2.5 e^−^ Å^−2^, deteriorating data and model quality in cryo-EM (Hattne *et al.*, 2018 ▸).

Following the initial MicroED studies on lysozyme and catalase demonstrating the potential for structural biology (Shi *et al.*, 2013 ▸; Nannenga, Shi, Leslie & Gonen, 2014 ▸; Nannenga, Shi, Hattne *et al.*, 2014 ▸), several other structures were determined from 3D protein crystals including various membrane proteins and ligand-bound complexes [Fig. 1 ▸(*b*)] (Yonekura *et al.*, 2015 ▸; de la Cruz *et al.*, 2017 ▸; Clabbers *et al.*, 2017 ▸; Liu & Gonen, 2018 ▸; Purdy *et al.*, 2018 ▸; Yonekura *et al.*, 2019 ▸; Wolff *et al.*, 2020 ▸; Clabbers *et al.*, 2020 ▸; Martynowycz *et al.*, 2020 ▸; Martynowycz, Shiriaeva *et al.*, 2021 ▸). In diffraction, the phase information is lost which is one of the classical problems in crystallography. In electron crystallography of 2D protein crystals, phases were provided by imaging and combined with higher-resolution intensities from diffraction (Glaeser & Downing, 1993 ▸; Fujiyoshi, 1998 ▸). However, extracting phase information from 2D projections of 3D crystals in cryoEM is much more challenging (Nederlof, Li *et al.*, 2013 ▸). In macromolecular crystallography, molecular replacement is the most commonly used method to solve novel protein structures, using phase information of a homology model. Molecular replacement was first implemented successfully in the electron crystallography structure of aqua­porin-0, a novel structure that was phased using the structure of AQP1 (Gonen *et al.*, 2004 ▸). Recently, a novel structure was presented of the metalloenzyme R2lox using MicroED (Xu *et al.*, 2019 ▸), and the TIR domain of the signaling adapter protein MyD88 was solved from higher-order crystalline assemblies showing several structurally remodeled regions compared with monomeric crystal and solution structures (Clabbers *et al.*, 2021 ▸).

Owing to the similarities in MicroED and MX data collection strategies using continuous rotation (Arndt & Wonacott, 1977 ▸; Dauter, 1999 ▸; Nannenga, Shi, Leslie & Gonen, 2014 ▸), standard crystallographic software originally intended to process MX data also work with MicroED data (Nannenga, Shi, Leslie & Gonen, 2014 ▸; Leslie, 2006 ▸; Kabsch, 2010 ▸; Winter *et al.*, 2018 ▸; Hattne *et al.*, 2015 ▸; Clabbers *et al.*, 2018 ▸). There are however some differences that need to be considered. Most notably, X-ray crystallography data are typically collected using 12 keV X-ray photons and have a highly curved Ewald sphere construction and large scattering angle [Fig. 2 ▸(*a*)]. In electron diffraction, the wavelength of high-energy electrons is much shorter resulting in an almost flat Ewald sphere and a small scattering angle [Fig. 2 ▸(*b*)]. This has several implications for MicroED: (1) reflections on each frame are from a virtually planar slice through reciprocal space and higher-order Laue zones are typically not observed; (2) Friedel pairs can sometimes be observed on the same frame; and (3) the sample-to-detector distance is linearly correlated to the unit-cell dimensions and refinement of those parameters should be decoupled. Therefore, MicroED data typically do not have sufficient information for indexing the diffraction patterns in all three crystallographic dimensions from a single frame, whereas this is feasible for X-ray diffraction data that clearly show the lunes of the higher-order Laue reflections (Fig. 2 ▸). Instead, for MicroED, a wedge of reciprocal data is required for successful indexing without *a priori* knowledge of unit-cell dimensions and symmetry. Around 20° of rotation typically suffice per crystal (Nannenga, Shi, Leslie & Gonen, 2014 ▸).

## 4. MicroED sample preparation and focused ion-beam milling

In cryo-EM sample preparation, biological specimens are typically deposited onto an electron microscope (EM) grid, any excess liquid is removed and the sample is then rapidly vitrified (Adrian *et al.*, 1984 ▸). MicroED sample preparation of protein 3D crystals takes a similar approach, using back-side blotting in a humidity- and temperature-controlled setup, followed by plunge-freezing the grid in liquid ethane (Fig. 3 ▸) (Shi *et al.*, 2016 ▸). Crystallization is an essential step in crystallography and often complicates structure determination by failing to grow large enough crystals for X-ray diffraction (Nave & Hill, 2005 ▸; Holton & Frankel, 2010 ▸; Sanishvili *et al.*, 2011 ▸). Crystallization screens that do not yield any sizeable crystal hits are typically discarded but may contain small macromolecular crystals that can be studied by cryoEM (Stevenson *et al.*, 2014 ▸; Calero *et al.*, 2014 ▸). Standard crystallization routines can be geared towards optimizing the conditions for growing smaller crystals by seeding or changing protein and precipitant concentrations to initiate a larger number of nucleation sites (Beale *et al.*, 2019 ▸). Even more so, MicroED can be used to study biomolecules that naturally assemble into thin microcrystal filaments, for example short peptide fragments aggregating and assembling into protofibrils related to Parkinson’s and Alzheimer’s disease (Rodriguez *et al.*, 2015 ▸; Sawaya *et al.*, 2016 ▸), or assembly formation of proteins involved in signal transduction during innate immune response (Clabbers *et al.*, 2021 ▸).

Alternatively, if crystals are too large, these can be fragmented into smaller crystals before grid preparation to yield high-quality structures (de la Cruz *et al.*, 2017 ▸). Recently, several groups reported the preparation of thin crystalline lamellae for MicroED using focused ion beam (FIB) milling and scanning electron microscopy (SEM) (Duyvesteyn *et al.*, 2018 ▸; Li *et al.*, 2018 ▸; Zhou *et al.*, 2019 ▸; Martynowycz *et al.*, 2019*a* ▸). Here, vitrified EM grids with protein crystals are loaded into a dual-beam FIB/SEM, suitable crystals are located using the electron beam and are thinned using a high-current gallium ion beam to a lamella of suitable thickness (Fig. 3 ▸) (Martynowycz & Gonen, 2021*b* ▸). Radiation damage to the crystal lamellae during the milling process can be reduced by pre-coating the grids with a thin layer of platinum, and by polishing at a lower current in the final thinning steps (Martynowycz *et al.*, 2019*b* ▸). Membrane proteins have both hydro­philic and hydro­phobic regions on their surface and require additional stabilization with detergents or lipids during purification and crystallization. Crystallization of membrane proteins is carried out in the presence of detergents or a lipidic environment such as bicelles of lipidic cubic phase (LCP) (Fig. 6) (Landau & Rosenbusch, 1996 ▸; Faham & Bowie, 2002 ▸; Cherezov, 2011 ▸; Ujwal & Abramson, 2012 ▸). The high viscosity of these lipidic mesophases imposes additional challenges for sample preparation. These challenges can be addressed by dilution or dissolving of the lipidic matrix (Zhu *et al.*, 2020 ▸; Martynowycz, Shiriaeva *et al.*, 2021 ▸), and by removing the surrounding lipid layers by FIB milling (Polovinkin *et al.*, 2020 ▸; Martynowycz *et al.*, 2020 ▸; Martynowycz, Shiriaeva *et al.*, 2021 ▸). It is important to keep the crystals hydrated during sample preparation by adding mother liquor on top of the crystal drops to reduce evaporation, minimizing lipid exposure to environmental air and keeping the grid preparation chamber at over 90% humidity (Martynowycz *et al.*, 2020 ▸).

Protein microcrystals that are too large, or embedded in a thick layer of solvent or vitreous ice, can be milled into thin crystalline lamella of a suitable thickness for MicroED. A concern that has historically hampered some of the initial enthusiasm on electron diffraction is multiple elastic electron scattering, or dynamical scattering (Cowley & Moodie, 1957 ▸; Fujiwara, 1959 ▸). Dynamical scattering affects the measured intensities and thus breaks the first-order kinematic approximation used in structure refinement. Early publications suggested intensities could be treated as quasi-kinematical for hydrated 3D microcrystals of catalase as thick as 150 nm (Dorset & Parsons, 1975*a* ▸). Another report mentions the negligible influence of dynamical effects at 120 kV for a 4.5 nm thin 2D crystal of bacteriorhodopsin based on experimental evidence from small intensity differences measured between Friedel pairs (Glaeser & Ceska, 1989 ▸). However, simulations suggested an upper limit of about 10 to 20 nm at 100 kV based on estimated Friedel differences of multi-layered bacteriorhodopsin crystals (Glaeser & Downing, 1993 ▸). Later, a maximum thickness of 100 nm at 200 kV was suggested based on multislice calculations of 3D lysozyme crystals (Subramanian *et al.*, 2015 ▸). These simulations however assume a stationary crystal, aligned and stacked along a major zone-axis, and ignore the contribution of disordered bulk solvent and inelastic scattering (Latychevskaia & Abrahams, 2019 ▸).

Any systematic investigation of ideal crystal thickness for MicroED was complicated until recently. Using FIB milling, it was observed that the highest resolution was attained at 185 nm out of four proteinase K lamellae at 200 kV, with diminishing returns by going to either thicker or thinner crystal lamellae (Zhou *et al.*, 2019 ▸). Furthermore, higher resolution and better model statistics were observed for thinned lamellae of microcrystals compared with data from thin nanocrystals that were not subjected to milling (Beale *et al.*, 2020 ▸). An optimal specimen thickness for cryo-EM was systematically investigated by thinning several proteinase K lamellae to a thickness corresponding to multiples of the inelastic mean free path (MFP), *i.e.* the distance an electron travels through the specimen before it scatters inelastically (Martynowycz, Clabbers, Unge *et al.*, 2021 ▸). For example, at 300 kV the inelastic MFP for a typical protein crystal is approximately 317 nm. No large differences in structure quality were observed between lamellae with thicknesses ranging from 0.5 to 2× the MFP using 300 kV electrons. Owing to the increased inelastic scattering and absorption of high-energy electrons, only low-resolution reflections were observed at 3× MFP, whereas no signal was detected at any thickness beyond 4× MFP. A similar trend for the same multiples of the inelastic MFP was observed at 120 and 200 kV accelerating voltages (Martynowycz, Clabbers, Unge *et al.*, 2021 ▸). These results suggest that an ideal crystal has a sufficient number of unit-cell repeats to produce a strong enough signal, but should be thin enough to limit absorption and minimize dynamic effects. Although no attempts were made to quantify dynamical scattering, structures could be successfully determined from all lamellae that yielded integrated diffraction data, before the majority of scattered electrons are absorbed by the sample.

## 5. MicroED of small organic molecules

Electron crystallography is also a useful technique in structure determination of inorganics and organic small molecules (Dorset, 1995 ▸). In the 1970s, Fujiyoshi and colleagues developed minimal-dose (low-dose) procedures enabling them to resolve individual atoms from images of beam-sensitive metal–organic copper-phthalocyanine and Ag-TCNQ crystals, demonstrating that electron microscopy can be used effectively to image atoms from materials (Uyeda *et al.*, 1979 ▸, 1980 ▸; Fujiyoshi, 1998 ▸). Using material science procedures at higher dose rates, Hovmöller *et al.* (1984 ▸) determined the accurate 2D atomic positions of a metal oxide from projection images of an aligned 40 Å thin 3D crystal of five unit-cell layers. Soon after, 3D atomic positions could be reconstructed from images (Downing *et al.*, 1990 ▸; Dong *et al.*, 1992 ▸), and electron diffraction data could be used to accurately refine the atomic positions using the kinematic or dynamic approximations (Weirich *et al.*, 1996 ▸; Zandbergen *et al.*, 1997 ▸; Jansen *et al.*, 1998 ▸). These studies typically used carefully aligned crystals at a major zone axis, taking only a few projection images and diffraction patterns. Data collection and reconstruction in 3D from various zone axes at discrete tilt angles was successfully demonstrated for a quasicrystal approximant using imaging and diffraction (Zou *et al.*, 2003 ▸). This process was quite time-consuming, and data collection and processing were later automated combining coarse discrete tilt steps with beam precession or beam tilt (Kolb *et al.*, 2007 ▸; Zhang *et al.*, 2010 ▸). However, these protocols employ high exposures and diffraction using conditions that are generally not suitable for studying protein crystals. Although MicroED was originally developed as a cryo-EM method using flash-frozen and cryo-cooled radiation-sensitive protein crystals, depending on the characteristics of the sample the experiments can be conducted at ambient temperature as well. In MicroED, diffraction data are rapidly collected under low-dose conditions of randomly oriented crystals that are continuously rotated [Fig. 4 ▸(*a*)] (Nannenga, Shi, Leslie & Gonen, 2014 ▸; Shi *et al.*, 2016 ▸). This facilitates fast data collection and structure determination at atomic resolution of beam-sensitive small organic molecules with a turnover that is competitive with X-ray diffraction (Jones *et al.*, 2018 ▸; Gruene *et al.*, 2018 ▸). Sample preparation is relatively straightforward, dry powders can be crushed or ground and directly applied to a standard EM grid (Fig. 4 ▸) (Gallagher-Jones *et al.*, 2018 ▸). During data acquisition, the grid is screened and individual crystals are selected for MicroED data collection, to distinguish different compounds from a mixture of crystals [Fig. 4 ▸(*b*)] (Jones *et al.*, 2018 ▸). This opens up many possibilities in the study of, for example, natural products (Ting *et al.*, 2019 ▸; Dick *et al.*, 2019 ▸; Halaby *et al.*, 2021 ▸; Kim, Xue *et al.*, 2021 ▸; Kim, Ohashi *et al.*, 2021 ▸), polymers (Ueda *et al.*, 2021 ▸), polymorphism (Broadhurst *et al.*, 2020 ▸) and the characterization of pharmaceutical compounds using MicroED (Jones *et al.*, 2018 ▸; Gruene *et al.*, 2018 ▸; Clabbers *et al.*, 2019 ▸; Bruhn *et al.*, 2021 ▸).

## 6. MicroED in fragment screening and drug discovery

Structural biology plays an important role in drug discovery. Structure-based drug design uses high-quality structural models of proteins that provide a detailed insight into their function, guiding the design of novel drugs (Blundell *et al.*, 2002 ▸). High-throughput fragment-based screening uses large libraries of compounds that are screened for potential binding interactions with a target protein (Hajduk & Greer, 2007 ▸). MicroED has several aspects that can make it attractive as a method for drug-discovery studies. For example, in MicroED, much smaller protein crystals can be used for structure determination. Furthermore, the smaller crystal volume has the advantage that any perturbations to the sample can be introduced much faster, such as rapid vitrification and efficient ligand soaking with the incorporation of small compounds at high occupancy (Fig. 5 ▸) (Martynowycz & Gonen, 2021*a* ▸). Additionally, FIB milling of soaked microcrystals into thin lamellae may have the advantage that no background noise is included from any excess unbound ligand in solution (Martynowycz & Gonen, 2021*a* ▸). High-throughput ligand incorporation and screening is well established in X-ray crystallography, and although the same level of throughput and automation is not yet standard in MicroED, it enables accessible in-house screening of possible ligand-binding interactions on a conventional TEM (Clabbers *et al.*, 2020 ▸).

Several examples demonstrate drug-discovery efforts using MicroED. Drug binding interactions were investigated by MicroED aiming to resolve the inhibitor bevirimat (BVM) bound to the C-terminal domain of the HIV Gag protein fragment [Fig. 5 ▸(*b*)] (Purdy *et al.*, 2018 ▸). Here, the apo structure without the drug was determined at 3.0 Å using MicroED, showing a six-helical bundle arrangement of the CTD-SP1 hexamer with an empty pore. The structural model in the presence of BVM was determined at 2.9 Å resolution, and the difference density was observed in the pore where the inhibitor is expected to bind. However, a unique binding pose could not be assigned based on the MicroED data alone (Purdy *et al.*, 2018 ▸). This was further complicated by the location of the binding position along one of the crystallographic symmetry operators at the center of the homo-multimer. Another report discusses the novel structure of an R2-like ligand-binding oxidase at 3.0 Å resolution (Xu *et al.*, 2019 ▸). The ligand, a long saturated fatty acid chain, was not observed from the difference map. However, this study was not specifically aimed at resolving the bound ligand, and low occupancy and flexibility of the ligand may have contributed to the absence of any ligand density. Interestingly, a remodeling of both shape and potential distribution of the binding pocket was observed from the structural model, indicating a possible different substrate-binding specificity compared with previously characterized homologs by X-ray diffraction (Xu *et al.*, 2019 ▸). Recently, MicroED was successfully used to resolve ligand-binding interactions at 2.5 Å resolution of the clinical drug acetazolamide to the active site of human carbonic anhydrase isoform II [Fig. 5 ▸(*c*)] (Clabbers *et al.*, 2020 ▸). The apo structure confirmed the unbound state of the protein, whereas the difference map in the drug-bound complex was used to fit and refine the position of the inhibitor, accurately revealing the underlying interactions involved in ligand binding (Clabbers *et al.*, 2020 ▸).

## 7. Structure determination of membrane proteins by MicroED

MicroED was originally developed for studying membrane protein structures. This is because membrane proteins are natively embedded in the lipid bilayer and are therefore hard to express, they purify in small quantities and, if lucky, form crystals that are frequently too small for investigation by X-ray crystallography. However, these thin crystals, typically around 200 nm, are ideal for MicroED. Several membrane proteins have been determined to date with MicroED. For example, the calcium-dependent ATPase solved from thin 3D crystals provided information on the charged state of atoms, which holds important implications for membrane biophysics (Yonekura *et al.*, 2015 ▸). The MicroED structure of the non-selective NaK ion channel was solved by MicroED from crystals containing only ∼1000 diffracting units (Fig. 6 ▸) (Liu & Gonen, 2018 ▸). The sample was crystallized in detergent using a sparse matrix screen and crystals appeared as granular aggregates. The crystal slurry was prepared using standard vitrification protocols similar to those of soluble protein crystals; crystals were easy to pipette and the excess solution was removed by blotting (Fig. 3 ▸). MicroED data were collected using continuous rotation, and data from 11 crystals were merged to obtain a complete dataset and a fully refined structure at 2.5 Å resolution (Liu & Gonen, 2018 ▸). A new transient state was captured in which a partially hydrated Na^+^ ion was observed at the entrance of the channel selectivity filter. A mechanism was proposed by which the side chain of Asn68 could rotate to open and close the channel pore like an iris, pulling the sodium ion deeper into the channel. The proposed mechanism may be universal among other Na^+^-conducting channels in symmetric assemblies. This work suggests that MicroED can provide information about unusual conformations and sparsely populated states of proteins, which may be harder to capture using large crystals.

Recently, MicroED was used to determine the structure of the novel K12E mutant mammalian mitochondrial voltage-dependent anion channel (mVDAC) (Martynowycz *et al.*, 2020 ▸). The wild-type ion channel was previously studied by X-ray crystallography (Bayrhuber *et al.*, 2008 ▸; Meins *et al.*, 2008 ▸; Ujwal *et al.*, 2008 ▸; Choudhary *et al.*, 2014 ▸; Schredelseker *et al.*, 2014 ▸). However, crystallization efforts of K12E mutant yielded only small crystals that could not be targeted by traditional X-ray crystallography techniques, and crystal-size optimization trials were unsuccessful. Therefore, MicroED was the most suitable technique for this type of sample. mVDAC was crystallized in lipid bicelles and thin plate-shaped microcrystals were located in the lipid layer using SEM and milled into thin lamellae using FIB/SEM to approximately 200 nm thickness (Martynowycz *et al.*, 2020 ▸). Merged data collected from three lamellae yielded 80% completeness at 3.1 Å resolution. The structure was solved by molecular replacement using the wild-type model. In addition to protein–protein interactions in the crystal packing, the lipids were found to likely mediate between the mVDAC monomers.

Furthermore, MicroED was successfully applied for studying G-protein coupled receptors from crystals grown in the LCP by solving the structure of the adenosine A_2A_ receptor (Fig. 6 ▸) (Martynowycz, Shiriaeva *et al.*, 2021 ▸). The A_2A_ receptor is a significant pharmacological target for cardiovascular and neurodegenerative diseases (Fredholm *et al.*, 2011 ▸; Franco & Navarro, 2018 ▸), and a member of the G-protein coupled receptor (GPCR) family – a large family of membrane proteins that play a key role in signal transduction inside the cell in response to signaling molecules and environmental changes. Crystallization of GPCRs is typically carried out in the LCP (Batyuk *et al.*, 2016 ▸). The high viscosity of the LCP is an obstacle to sample preparation and MicroED data collection. To overcome this issue, the LCP was converted into the sponge phase by the addition of PEG400 in a humidity-controlled environment. Subsequently, microcrystals were located on a TEM grid using SEM and FIB milled into thin lamellae ∼200 nm thick. MicroED data were collected from a single lamella over a wedge of approximately 70°. The total diffracting volume was less than 1 µm^3^ (0.2 × 2 × 2 µm), which is lower than any other method used for GPCR structure determination to date. The total exposure used at 300 kV was only about 2 e^−^ Å^−2^, equivalent to ∼7.4 MGy (Baker & Rubinstein, 2010 ▸), which minimized radiation damage. The adenosine A_2A_ receptor lamella yielded 80% completeness and a high-quality map and structural model to 2.8 Å resolution. The overall structure of A_2A_AR is consistent with previous reports (Liu *et al.*, 2012 ▸; Batyuk *et al.*, 2016 ▸). MicroED allowed the ligand ZM241385 in the orthosteric pocket and four surrounding cholesterol molecules bound to the receptor on the extracellular side to be resolved [Figs. 5 ▸(*e*) and 6 ▸].

## 8. Future perspectives

MicroED offers opportunities for solving structures at atomic resolution using crystals a millionth the size of those used for X-ray crystallography. For small molecules, this means that structures can be obtained directly from powders using femtogram amounts of material within minutes. For radiation-hard materials where high doses can be used, the method can likewise deliver structures rapidly and with little effort. Proteins, however, are much more difficult to study as an extremely low electron dose must be used coupled with cryogenic cooling to protect the sample from the vacuum and exposure. Faster cameras make reduce data collection times so the total exposure on the sample can be further minimized. By perfecting the sample thickness, matching the correct acceleration voltage, and coupling to a fast and highly sensitive camera recent studies demonstrated subatomic structure determination even for proteins and phase determination by *ab initio* methods (Martynowycz, Clabbers, Hattne & Gonen, 2021 ▸). While the development of MicroED procedures is still ongoing, the growth in the user base means that nationally funded centers for MicroED are needed. When equipment can be accessed and expertise become available freely, the method will gain more momentum and benefit more researchers in diverse fields including structural biology of macromolecules, materials science, semiconductors, natural products, and chemistry for drug discovery and development.
